# Substantial Conformational Change Mediated by Charge-Triad Residues of the Death Effector Domain in Protein-Protein Interactions

**DOI:** 10.1371/journal.pone.0083421

**Published:** 2013-12-31

**Authors:** Edward C. Twomey, Dana F. Cordasco, Stephen D. Kozuch, Yufeng Wei

**Affiliations:** 1 Institute of NeuroImmune Pharmacology, Seton Hall University, South Orange, New Jersey, United States of America; 2 Department of Chemistry and Biochemistry, Seton Hall University, South Orange, New Jersey, United States of America; 3 Integrated Program in Cellular, Molecular, and Biomedical Studies, Columbia University, New York, New York, United States of America; Indian Institute of Science, India

## Abstract

Protein conformational changes are commonly associated with the formation of protein complexes. The non-catalytic death effector domains (DEDs) mediate protein-protein interactions in a variety of cellular processes, including apoptosis, proliferation and migration, and glucose metabolism. Here, using NMR residual dipolar coupling (RDC) data, we report a conformational change in the DED of the phosphoprotein enriched in astrocytes, 15 kDa (PEA-15) protein in the complex with a mitogen-activated protein (MAP) kinase, extracellular regulated kinase 2 (ERK2), which is essential in regulating ERK2 cellular distribution and function in cell proliferation and migration. The most significant conformational change in PEA-15 happens at helices α2, α3, and α4, which also possess the highest flexibility among the six-helix bundle of the DED. This crucial conformational change is modulated by the D/E-RxDL charge-triad motif, one of the prominent structural features of DEDs, together with a number of other electrostatic and hydrogen bonding interactions on the protein surface. Charge-triad motif promotes the optimal orientation of key residues and expands the binding interface to accommodate protein-protein interactions. However, the charge-triad residues are not directly involved in the binding interface between PEA-15 and ERK2.

## Introduction

Protein conformational changes are fundamental biochemical phenomena in protein-protein interactions, and are crucial for mediating protein functions. Death effector domain (DED) belongs to a superfamily of death structural domains that primarily promote homotypic domain-domain interactions in the tumor necrosis factor (TNF)-mediated apoptotic cascade activated by the formation of the death-inducing signaling complex (DISC) [1]. Subfamilies in the death structural domain superfamily, including the death domain (DD), DED, caspase recruitment domain (CARD), and pyrin domain (PYD), are sequentially homologous and fold similarly into a six-helix (α1–α6) bundle, but are differentiated by domain-domain interactions. DED structural domains possess unique surface features, one of them being the charge-triad, a hydrogen-bonded network between side chains of three charged residues, D/E-RxDL (x = any residues), located on helices α2 and α6 [1], [2]. Despite the fact that the charge-triad is highly conserved among almost all DED proteins, the role of the charge-triad is still poorly understood.

DED proteins are also reported to interact with various proteins in distinct biological pathways to modulate biological processes in addition to apoptosis [2]. The small non-catalytic, DED-containing protein, phosphoprotein enriched in astrocytes, 15 kDa (PEA-15) interacts with Fas-associated death domain (FADD) protein DED, preventing the recruitment of procaspase-8 into the DISC and blocking TNFα-mediated apoptosis pathway [3]. PEA-15 also interacts with mitogen-activated protein (MAP) kinases, extracellular regulated kinase (ERK) 1 and 2, with high affinity, inhibiting ERK-dependent cell proliferation and migration through the localization of ERK1/2 to the cytosol [4], [5]. The structure of PEA-15 in the free form was first determined using NMR spectroscopy (PDB ID 1N3K). This NMR model and mutagenesis study suggested that, D^74^, one of the charge-triad residues, of the PEA-15 DED was located at the binding interface with ERK2 [6]. However, this model has been largely invalidated by our recent NMR dynamics study [7] and by recent crystal structures of the PEA-15/ERK2 complexes [8]. Our NMR dynamic data suggested that PEA-15 utilizes residues on helices α5 and α6 to interact with ERK2, whereas charge-triad residues are not directly involved in binding ERK2 [7]. We additionally refined the PEA-15 DED structure in the free form (PDB ID 2LS7), in which the charge-triad motif, D^19^-R^72^-D^74^, and a number of other electrostatic and hydrogen bonding interactions are clearly identifiable [9]. Our interaction model was recently confirmed by the crystal structures (PDB IDs: 4IZ5, 4IZ7, and 4IZA) showing that residues E^68^, R^71^, and P^73^ on PEA-15 directly interact with ERK2 through hydrogen bonding and hydrophobic effects, whereas the charge-triad residues, R^72^ and D^74^, are not involved in the binding interface, but serve to stabilize the orientation of the interface residues [8]. Although the crystal structures of the complexes clearly illustrate the binding interface between the two proteins, the conformation of the PEA-15 DED is not fully accessible due to the lack of electron density between helices α2 and α4 of the DED. The lack of electron density in this presumably well-folded domain agrees with our earlier NMR dynamic assessment of the PEA-15/ERK2 complex, and indicates that the PEA-15 DED possesses unusually flexible regions that could accommodate various interacting partners.

Based on the dynamic profiles of PEA-15 in the complex with ERK2, we previously proposed that the DED of PEA-15 undergoes a profound conformational change upon interaction with ERK2 [7], although NMR dynamic data did not provide direct evidence of the protein conformations, nor did they indicate any potential sites for the changes. In this study, using backbone residual dipolar couplings (RDCs) of PEA-15 in its free and ERK2-bound forms in two alignment media, we confirm that PEA-15 binding involves a substantial conformational change, and report the first direct experimental evidence for the regions of conformational change in PEA-15 DED upon binding with ERK2. NMR RDCs, which provide unique long-range orientational structural restraints of the molecule [10], are directly associated with protein conformation. In addition, our NMR data indicate that the charge-triad residues and polar surface contacts (i.e., hydrogen bonds and salt bridges) play more extensive roles in promoting protein-protein interactions. Delineating this conformational change and the potential functions of the charge-triad in various biological actions of PEA-15 could significantly impact our understanding of the regulations of MAP kinase and apoptosis pathways as well as the mechanism of drug resistance in cancer chemotherapy.

## Materials and Methods

### NMR spectroscopy

Uniformly ^2^H, ^13^C, and ^15^N-labeled full-length PEA-15 and natural abundance ERK2 were expressed as described previously [6]. Backbone residual dipolar couplings (RDCs) were measured for PEA-15 in its free form and in the complex with ERK2 in a 1∶1 ratio using the spin-state selective coherence transfer (S^3^CT) in-phase anti-phase (IPAP) technique [11] on a Bruker Avance 800 MHz NMR spectrometer equipped with a triple-resonance cryoprobe. Both free and complex RDC data were obtained from two independent alignment media, a 9 mg/ml filamentous bacteriophage pf1 (Asla-Biotech) [12], [13], and a 5% nonionic liquid crystalline solution formed by n-alkyl-poly(ethylene glycol) and denoted as C_12_E_5_ (Fluka), and *n*-hexanol (Aldrich) in water, with a C_12_E_5_/hexanol molar ratio of *r* = 0.85 [14]. All NMR experiments were performed at 298 K. Singular value decomposition (SVD) [15] and back-calculation of predicted RDC values were performed using the iDC toolkit [16].

### Structural Analysis

Surface polar interactions of PDB structures, 2LS7, 4IZ5, 4IZ7, 4IZA, and 2BBR, were performed using PyMOL version 1.5 (http://www.pymol.org) with customized python scripts. Small molecule charge-triad analogs were surveyed with a substructure search that included a carboxylate group, a guanidine group, and another carboxylate group using the WebCSD server [17] of the Cambridge Structural Database [18]. The small molecule analogs were then visualized and analyzed using CSD software Mercury version 3.0 [19].

## Results and Discussion

**Figure 1:**
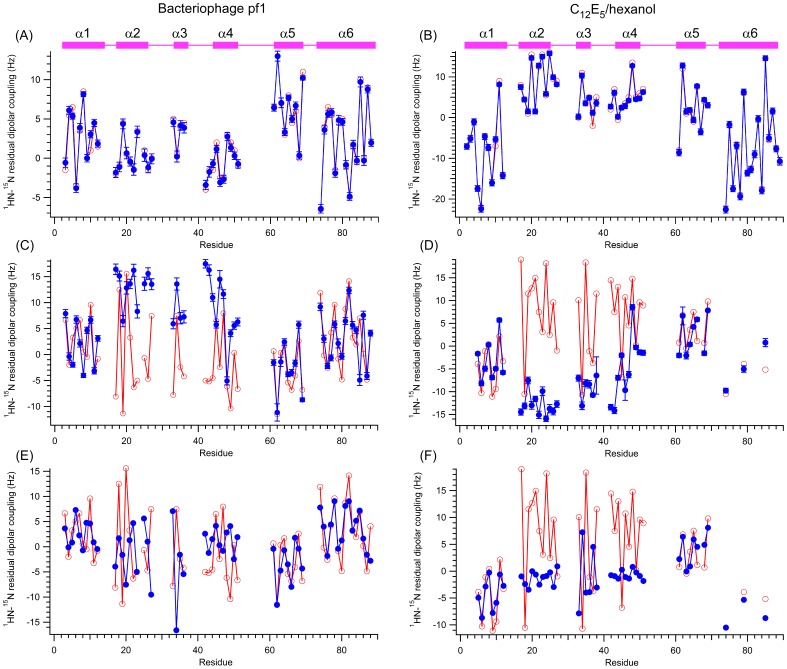
Comparison of experimentally-measured and predicted residual dipolar couplings (RDCs) of PEA-15 death effector domain (DED, residues 1–90). Experimental RDCs (red) were measured for the PEA-15 in the free (A, B) and ERK2-bound (C–F) forms in two alignment media: filamentous bacteriophage pf1 (A, C, E) and non-ionic C_12_E_5_/*n*-hexanol (B, D, F). Predicted RDCs (blue) were calculated from (A–D) the NMR model structure of free PEA-15 (2LS7) or (E, F) the ERK2-bound form of PEA-15 (4IZ7). The positions of the alpha-helices are indicated by pink bars. The free-form PEA-15 displays excellent agreement between the experimental and predicted RDC values throughout the DED sequence, while the ERK2-bound PEA-15 RDCs only reasonably agree in helices α1, α5, and α6 for both 2LS7 and 4IZ7, indicating a significant reorientation of helices α2, α3, and α4 in the PEA-15/ERK2 complex.

Full-length PEA-15 is bound tightly to ERK2 protein with a high affinity of *K*_d_ = 133±5 nM, and the phosphorylation states of both PEA-15 and ERK2 do not significantly affect the stability of the complex [5], [20]. Both dynamic light scattering [21] and our own NMR dynamics studies [7] revealed that PEA-15 and ERK2 form a strong 1∶1 complex of ∼57 kDa in solution. Under the same NMR solution conditions, we measured the RDC data for the full-length PEA-15 in its free-form and in complex with unphosphorylated ERK2. The singular value decomposition (SVD) analysis [15], using the free-form RDC data for DED residues 1–90, on the free-form structure, PDB ID 2LS7 [9], yielded a perfect match between the experimental and predicted ^1^H-^15^N RDC values for both alignment media (Figures 1a and 1d). However, using the complex RDC data on the same 2LS7 structure did not yield any reasonable fit (see Figure S1), suggesting that significant reorientation had occurred among the six helices of the PEA-15 DED upon complex formation, and therefore, no reasonable agreement between the predicted and experimental RDC values could be obtained.

Our earlier NMR dynamic study on the PEA-15 and ERK2 complex suggested that the PEA-15 DED tumbles as two distinct segments in the complex, one segment composed of helices α1, α5, and α6, possessing a slow tumbling rate with a correlation time, τ*_c_* ∼15 ns, and the other segment, composed of helices α2, α3 and α4, tumbling at a fast rate, with τ*_c_* ∼11 ns [7]. The dynamic data indicated that the relative rotations could happen between these two segments upon complex formation. To assess this possibility, using the complex RDC values of the slow (α1, α5, and α6) and fast tumbling (α2, α3 and α4) segments, we performed SVD analyses against the free-form structure of PEA-15. In each media, SVD analysis of the slow tumbling segment (α1, α5, and α6) fit reasonably well, whereas the fast tumbling segment (α2, α3, and α4) did not generate any reasonable fit (see Figure S1). These results strongly suggest that, upon formation of the PEA-15/ERK2 complex, the helices α1, α5, and α6 of the PEA-15 DED maintain their free-form conformation to a large extent, whereas helices α2, α3, and α4 significantly change their conformations and reorient themselves with respect to helices α1, α5, and α6. Using the alignment tensors obtained from the slow tumbling segment, we back-calculated a set of predicted RDCs for the bound state using the free-form structure, 2LS7, and compared with the experimental RDCs (Figures 1b and 1e). In both alignment media, the predicted bound-form RDCs agreed well with the experimental values for helices α1, α5, and α6, whereas the predicted and experimental values deviated substantially for helices α2, α3, and α4. The fact that the fast tumbling segment cannot be fitted into an alignment tensor for either alignment media indicates that the complex structure cannot be formed by rigid-body rotations between the two segments, implying complex motions in the fast tumbling segment.

Recently, various complex structures between PEA-15 and ERK2 have been reported [8], including a full-length PEA-15 in complex with the T185E ERK2 mutant (PDB ID 4IZ5), PEA-15 DED (residues 1–96) in complexes with unphosphorylated ERK2 (PDB ID 4IZ7) and dual-phosphorylated (pT185 and pY187) ERK2 (PDB ID 4IZA). We also tried to fit the experimental RDC data in the complex to the recently reported crystal structure, 4IZ7, which is closest to our experimental conditions and has the highest resolution among the three structures. The back-calculated PEA-15 RDCs, using 4IZ7 coordinates, agreed reasonably with the experimental data only in helices α1, α5, and α6 for the ERK2-bound form, whereas the predicted and experimental RDCs showed poor agreement for helices α2, α3, and α4 in both media (Figure 1E and 1F, and Figure S2), although the disagreement was much less compared to the fit to the free-form structure. The RDC data indicate that the crystal structures of the PEA-15/ERK2 complex differ considerably from the solution conformations, particularly in the fast tumbling segment composed of helices α2, α3, and α4. The NMR dynamic data indicated an overly flexible DED in the PEA-15/ERK2 complex, particularly in the α2, α3, and α4 region. This conformational flexibility contributes to the discrepancy between the solution and the crystal structures as well as the disagreement between the experimental and predicted RDC data, as RDCs contain rich information on slow dynamics reflecting conformational fluctuations on a millisecond to second timescale [22].

The charge-triad is a prominent structural feature in almost all death effector domains [1], [2]. The role of the charge-triad, however, is poorly understood. Although it has been suggested that the charge-triad motif is at the binding interface between the DED and other proteins [6], that model has been largely invalidated by NMR dynamic studies and crystal structures. To understand the functions of the charge-triad, and potentially other surface electrostatic and hydrogen bonding interactions, in mediating protein-protein interactions involving DEDs, we examined the available DED structures, including the high-definition NMR structure of PEA-15, 2LS7 [9], the NMR structure of FADD residues 1–191, 2GF5 [23], and the crystal structure of the vFLIP MC159 protein, 2BBR [24] and 2F1S [25], together with the most recent crystal structures of PEA-15 in complexes with ERK2, 4IZ5, 4IZ7, and 4IZA [8]. We have also surveyed the Cambridge Structural Database (CSD) [18] for crystal structures of small organic molecules that contain hydrogen bonding networks formed by carboxylate, guanidine, and another carboxylate, which mimics the charge-triad motif observed in DEDs, to assess the ordinariness and potential strengths of the charge-triad hydrogen bonding network. Electrostatic and hydrogen bonding interactions in terms of bond strengths in small molecules are well-documented, which can be used to estimate hydrogen bonding strengths involving similar functional groups in biomolecules. Most importantly, inferred from small molecule studies in both gas and condensed phases, these electrostatic and hydrogen bonding interactions in the proteins reduce the barriers between multiple minima in a large volume, resulting in an easy transition among several conformational states controlled by the movement of the charges and protons along the direction of the hydrogen bonding network [26].

**Figure 2:**
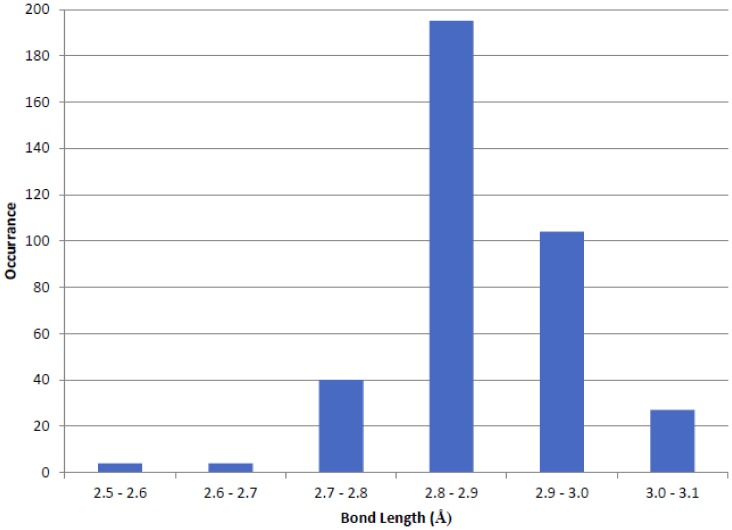
Distribution of distances between nitrogen and oxygen atoms involved in N-H…O hydrogen bonds in small molecule structures containing intermolecular carboxylate-guanidine-carboxylate (charge-triad mimic) motifs surveyed in the Cambridge Structural Database (CSD). A total of 109 structures that possess the charge-triad mimics were extracted from the CSD and hydrogen bonding patterns analyzed to generate the histogram. Only intermolecular hydrogen bonds formed between COO^−^…H-N^+^-H…^−^OOC were counted in the histogram.

**Figure 3:**
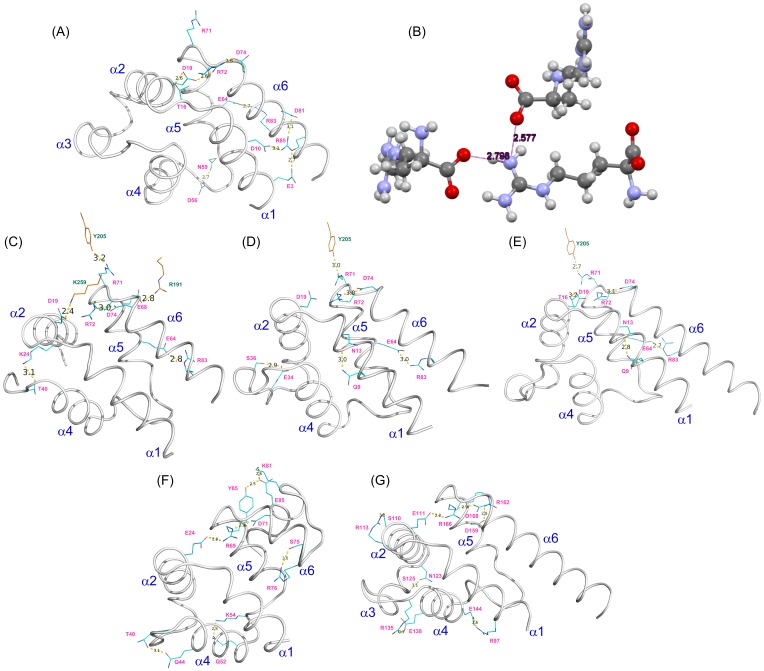
Electrostatic and hydrogen bonding interactions between charged and polar amino acid side chains. (A) PEA-15 DED in the free form (PDB ID 2LS7). (B) L-arginine (CSD Refcode TAQBIY). (C) Full-length PEA-15 complexed with T185E ERK2 (PDB ID 4IZ5). (D) PEA-15 DED complexed with unphosphorylated ERK2 (PDB ID 4IZ7). (E) PEA-15 DED complexed with dual phosphorylated ERK2 (PDB ID 4IZA). (F) MC159 protein DED1 (PDB ID 2BBR). (G) MC159 protein DED2 (PDB ID 2BBR). The six helices, α1–α6, are labeled on each DED structure, with residues forming hydrogen bonds represented as a stick model (cyan color with pink labels), and heavy atom distances (in Å) shown between the two residues. Residues from ERK2 (C–E) are colored in orange with green labels.

The survey of the CSD yielded 109 structures that contain the COO^−^…H-N^+^-H…^−^OOC charge-triad mimic, with heavy atom distances between 2.577 Å and 3.06 Å and a median distance of 2.862 Å. The distribution of hydrogen bonding distances are plotted in Figure 2. The majority of the N-H…O hydrogen bonding distances fell within the expected range of 2.7–3.0 Å, although both relatively short (<2.6 Å) and long (>3.0 Å) hydrogen bonds were observed. The hydrogen bonds for the charge-triad motif observed in PEA-15 and FADD DEDs were fairly short in comparison with small molecules. D^19^-R^72^ and R^72^-D^74^ heavy atom distances were 2.67±0.08 Å and 2.72±0.08 Å, respectively, in PEA-15 in its free-form (2LS7, Figure 3A), and E^19^-R^72^ and R^72^-D^74^ heavy atom distances were 2.65±0.03 Å and 2.76±0.09 Å, respectively, in FADD DED (2GF5). These distances are comparable to a recent crystal structure of the L-arginine amino acid (CSD Refcode TAQBIY) [27], in which a charge-triad mimic structure is formed between two carboxylate groups (p*K_a_*∼2.2) and one guanidine group (p*K_a_*∼12.5) with N-H…O hydrogen bonding distances of 2.577 Å and 2.798 Å (Figure 3B), representing one of the shortest N-H…O hydrogen bonds among small organic molecules in the CSD [28]. In the ERK2-bound forms, PEA-15 DED maintained only the R^72^-D^74^ interaction, with a substantially increased heavy-atom distance of 3.0 Å (4IZ5, 4IZ7, and 4IZA, Figure 3C–E), whereas D^19^ did not participate in the charge-triad interaction, but interacted with K^259^ of the ERK2 protein (4IZ5, Figure 3C). In the MC159 crystal structure, 2BBR, the charge-triad motif was also clearly visible for both DED1 and DED2 with heavy atom distances between 2.8 Å and 2.9 Å (Figure 3F–G), which are comparable to the median values among small molecules.

Although the charge-triad mimics in the small molecule crystal structures are possibly formed mostly by crystal packing, our survey of the CSD indicated that the hydrogen bonding network among the charge-triad functional groups can modulate the formation of more than 100 different structures and conformations with a wide range of hydrogen bonding distances. In addition, ionic hydrogen bonds formed between charged functional groups reduce the energy barrier between isomeric or conformational states, facilitating transitions between isomers or conformations [26]. On the DED surface, the charged and hydrogen bonding network is much more extensive than that in the small molecule crystals, as illustrated in PEA-15 and MC159 DEDs, and this extensive network is crucial for facilitating and controlling the protein conformational transitions as dictated by the biological processes. Therefore, as implied from the small molecule hydrogen bonding interactions, the charge-triad interactions on DEDs are not likely to provide additional energy in terms of stabilizing DED conformation. On the contrary, because the charge-triad is situated in a hinge position in between the α1-α5-α6 and α2-α3-α4 segments, the charge-triad residues, together with other charged and hydrogen bonding interactions on the DED, are important in maintaining necessary flexibility for controlled transitioning between conformational states in protein-protein interactions.

In addition to the canonical charge-triad motif, PEA-15 possesses many other electrostatic and hydrogen bonding interactions on the protein surface. Most notably, the E^64^-R^83^ interaction that holds helices α5 and α6 together was observed in both free PEA-15 and ERK2-bound forms (Figure 3A, C–E). A similar interaction was observed in MC159 (2BBR) DED1, in which a short hydrogen bond between Y^65^ and E^85^ was formed (Figure 3F). In free-form PEA-15, another network of hydrogen bonds, structurally mimicking the charge-triad motif, was identified among E^3^/D^10^-R^85^-D^81^ residues, which we have termed an “extended” charge-triad motif. This extended charge-triad effectively holds helices α1 and α6 together (Figure 3A). These polar interaction networks connect helices α1, α5, and α6, and dynamically reduce the flexibility in this region. In contrast, helices α2, α3, and α4 lack the inter-helical interactions, resulting in a flexible and dynamically complex region.

Based on our NMR data, we propose a model in which the characteristic charge-triad residues, D^19^-R^72^×D^74^L, which are positioned between the two dynamically distinct segments formed by α1-α5-α6 and α2-α3-α4 in the PEA-15 DED, mediate the conformational changes through the reorganization of an extensive polar interaction network. The more rigid region, comprised of helices α1-α5-α6 that is tightly held by several inter-helix hydrogen-bonding networks, including an extended charge-triad motif, E^3^/D^10^-R^85^-D^81^, undergoes little conformational change when interacting with ERK2, whereas the flexible region, consisting of helices α2-α3-α4, although not directly involved in binding, undergoes the most extensive change in conformation. The complex formation is also accompanied by rearrangement of the charge-triad interactions among D^19^-R^72^-D^74^, which, together with an extensive polar interaction network on the DED surface, maintains the essential flexibility of the DED backbone, allowing the necessary conformational changes that are energetically compensated for by the shuffling of electrostatic and hydrogen bonding networks upon interaction with other protein partners. A single point mutation, D74A on PEA-15, completely abolishes the ability to form the PEA-15/ERK2 complex [6]. Although D^74^ does not interact with ERK2, the mutation inevitably disrupts the interactive network among polar and charged residues on the DED surface, disabling the energetic compensation for the adoption of the necessary conformation to form a complex with ERK2. Therefore, the charge-triad motif mediates the protein-protein interactions by maintaining conformational flexibility of the DED.

It is notable that PEA-15, also referred to as phosphoprotein enriched in diabetes (PED-15), binds tightly to phospholipase D (PLD) isoforms 1 and 2, an interaction that ultimately leads to type II diabetes [29]. PEA-15 interacts with PLD1 through residues 1–24 with high affinity (*K*_D_ = 0.37±0.13 µM) [30]. A recent NMR study also suggested that PEA-15 utilizes a surface formed by residues in helices α1, α3, and α4, as well as flanking loops of α1–α2 and α3–α4 [31], including an extended charge-triad residue, E3. This observation supports the idea that surface polar interactions on DEDs can stimulate various conformational changes to form extensive binding interfaces upon complex formation with different partner proteins as proposed in our model. In the PEA-15/PLD1 complex, the extended charge-triad motif, E^3^/D^10^-R^85^-D^81^, appears to mediate the interaction with PLD1, although it serve to stabilize the α1-α5-α6 segment when interacting with ERK2.

Compared to death domain (DD) structures, DEDs are more versatile and inherently more dynamic and flexible structural domains. In the PDB, a handful of DD proteins [1] and protein complexes [32], [33] have reportedly crystalized, whereas, before the PEA-15/ERK2 complexes, only one DED protein, vFLIP MC159, was characterized by crystallography [24], [25] and no DED complex structures were available. DDs and DEDs possess no catalytic activities, and all their biological functions are asserted through modulating protein-protein interactions. The binding interfaces of these structural domains are quite extensive. Unlike DDs, which exclusively interact with other DDs [1], DEDs can interact with an array of proteins with distinct functions, shapes and sizes [2]. Therefore, it is important for DEDs to maintain extensive flexibility, allowing proper conformational changes to accommodate various proteins of different shapes and sizes, and to promote protein-protein interactions. Various polar interactions on the DED surface can facilitate and promote protein-protein interactions by maintaining the conformational flexibility and positioning key residues into optimal orientations to interact with other proteins. Disruption of certain polar interaction networks could eliminate the DED's ability to bind some protein partners due to reduced flexibility to adopt the necessary conformations.

## Supporting Information

Figure S1 **Singular value decomposition (SVD) analyses of RDC data (^1^H-^15^N) for PEA-15 DED in ERK2-bound form measured from two distinct media.**
(PDF)

Figure S2 **Singular value decomposition (SVD) analyses of RDC data (^1^H-^15^N) for PEA-15 DED in ERK2-bound form measured from two distinct media.**
(PDF)
