# Supplementary material for: Substantial Conformational Change Mediated by Charge-Triad Residues of the Death Effector Domain in Protein-Protein Interactions
Source: PLoS One. 2013 Dec 31;8(12):e83421. doi: 10.1371/journal.pone.0083421 (PMC3877032; doi:10.1371/journal.pone.0083421)
Supplement: Figure S1 — Singular value decomposition (SVD) analyses of RDC data (1H-15N) for PEA-15 DED in ERK2-bound form measured from two distinct media. (PDF) [file pone.0083421.s001.pdf]

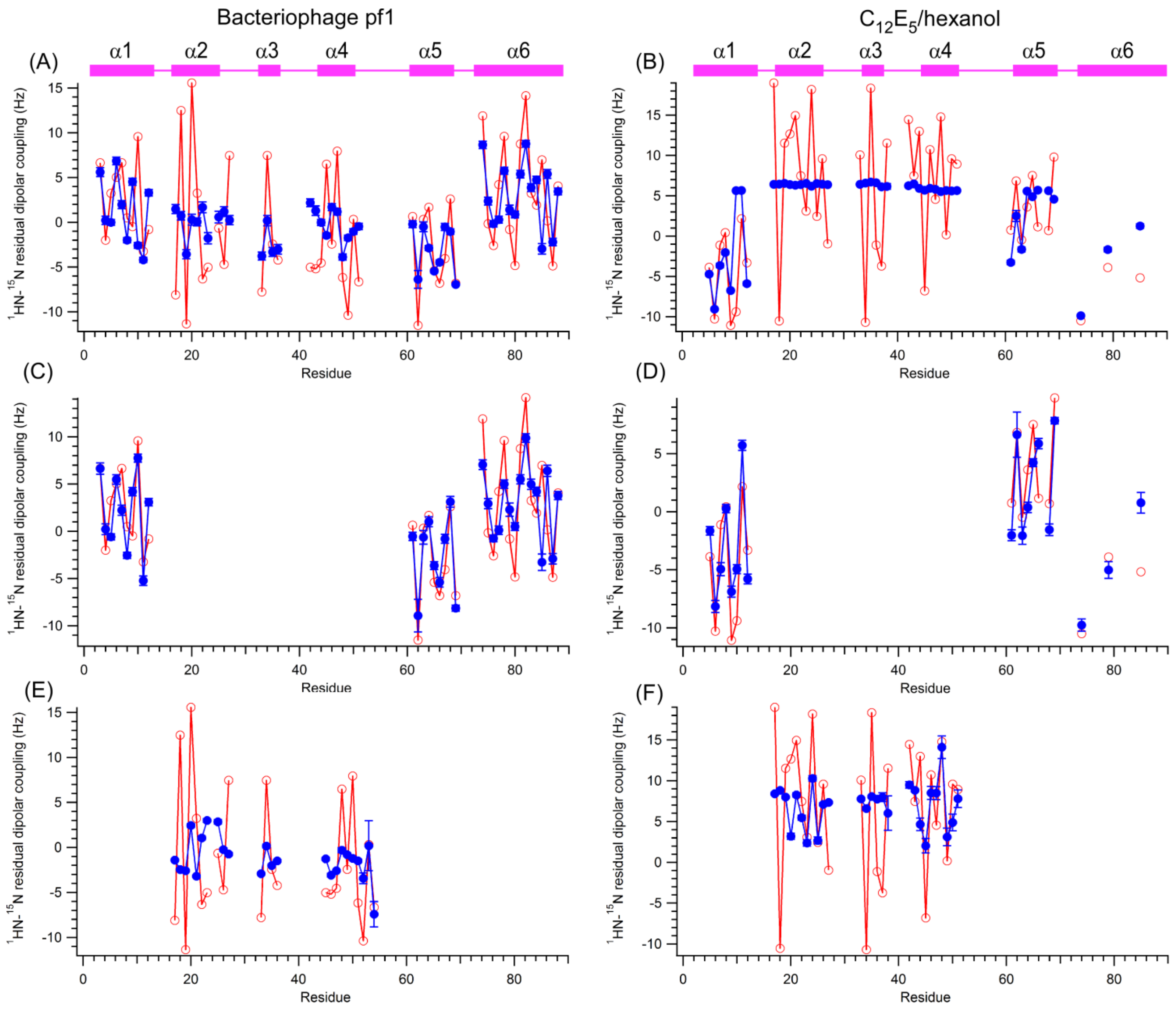

FIGURE S1 Singular value decomposition (SVD) analyses of RDC data (^{1}H-^{15}N) for PEA-15 DED in ERK2-bound form measured from two distinct media as labeled. The free-form NMR structure of PEA-15 DED, PDB 2LS7, was used in all analyses. The secondary α-helical structures are indicated on top of the plot. Experimental RDC values are shown in red and simulated values in blue. Error bars in simulated data indicate the standard deviation among all NMR models. (A) and (B) SVD analyses of RDC data for all DED residues (1-90); (C) and (D) SVD analyses of RDC data for helices α1, α5, and α6. A reasonable match between the predicted and experimental RDC values could be obtained in both media, and the order tensor matrix generated by the SVD was used in the back calculations of predicted RDC values as shown in the main manuscript. (E) and (F) SVD analyses of RDC data for helices α2, α3, and α4. The predicted and experimental RDCs do not match in this region, indicating a substantial rearrangement of α2, α3, and α4 relative to α1, α5, and α6.
